# In search of a perfect trait set: A workflow presentation based on the conservation status assessment of Poland's dendroflora

**DOI:** 10.1002/ece3.9979

**Published:** 2023-04-07

**Authors:** Sonia Paź‐Dyderska, Andrzej M. Jagodziński

**Affiliations:** ^1^ Institute of Dendrology Polish Academy of Sciences Kórnik Poland; ^2^ Department of Game Management and Forest Protection, Faculty of Forestry and Wood Technology Poznań University of Life Sciences Poznań Poland

**Keywords:** conservation status, data availability, ecological modeling, explanatory power, functional traits, plant organs

## Abstract

Considering the dynamically changing environment, we cannot be sure whether we are using the best possible plant functional traits to explain ecological mechanisms. We provide a quantitative comparison of 13 trait sets to determine the availability of functional traits representing different plant organs, assess the trait sets with the highest explanatory potential, and check whether including a higher number of traits in a model increases its accuracy. We evaluated the trait sets by preparing 13 models using similar methodology and responding to a research question: How do models with different sets of functional traits predict the conservation status of species? We used the dataset covering all woody species from Poland (*N* = 387), with 23 functional traits. Our findings indicate that what matters most for a trait set of high explanatory power is the precise selection of those traits. The best fit model was based on the findings of Díaz et al. (2016; The global spectrum of plant form and function, Nature, 529, 167‐171) and included only six traits. Importantly, traits representing different plant organs should be included whenever possible: Three of the four best models from our comparison were the ones that included traits of various plant organs.

## Graphical abstract

We found that including traits representing different plant organs improves the model output, but including more traits into a model does not necessarily increase the model's accuracy. Based on our results, we provide synthesized guidelines for other authors regarding the selection of the best possible traits for their research.

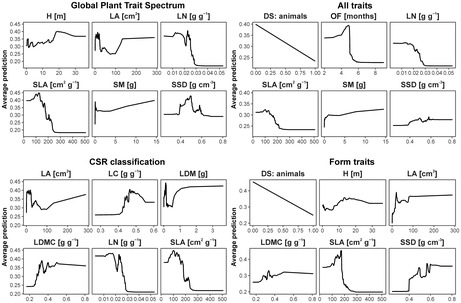

## 1 INTRODUCTION

Recognized, well‐covered traits are indispensable for large‐scale analyses that provide syntheses explaining processes driving ecosystems worldwide (Chave et al., 2018; Madani et al., 2018; Niinemets et al., 2015). Yet, processes taking place at different levels of life organization cannot always be explained using the same functional traits (Lanta et al., 2011; Messier et al., 2010), because the factors driving ecological processes also differ across spatial scales (Pearson & Dawson, 2003). Therefore, we should move toward a more even coverage of the functional traits representing the diverse organs and biological functions of a plant (Cornwell et al., 2019; Kleyer & Minden, 2015). As a consequence, we could overlook and ignore other, perhaps highly explanative traits (Moravcová et al., 2015). Moreover, covering less‐known traits with a higher number of measurements could reveal their hidden explanatory potential (Moravcová et al., 2015). Evenly covering different traits with measurements is the key to better‐balanced models: As long as data are missing, including poorly measured traits into models remains unjustified (Cornwell et al., 2019).

While trait databases are becoming increasingly comprehensive and accessible, selecting the most suitable functional traits for analysis still remains an uncertain matter (Lefcheck et al., 2015; Mlambo, 2014; Rosado et al., 2013). Previous studies provided instructions on the best possible choice of traits, for example, by using “all traits that are important for the function of interest” (Petchey & Gaston, 2006). Expert recommendations are also available for more specific topics, such as the use of traits for climate change analyses (Green et al., 2022; Kühn et al., 2021). Authors of the recently published “Handbook of Trait‐Based Ecology” (de Bello et al., 2021) recommend to study only those traits with a clear and specific hypothesis on how the given trait affects the process studied (effect traits), or with a hypothesis on how the trait is affected by the process studied (response traits). They encourage researchers to use numerous traits during the explorative phase of the study, to find out which traits would work for a particular research question, rather than in the final phase of analyses, where (in most cases) we should go toward reducing the number of traits used. This point of view suggests that the more specific the research question, the easier the selection of the traits. What we found particularly interesting was the mention of using numerous traits at the initial stage of data analysis to explore which ones will suit our research aim in the best possible way. Yet, limitations such as rather narrow representation of species measurements found in databases (approximately 17% worldwide; (Cornwell et al., 2019), uneven geographic coverage (biased towards the Global North; (Kleyer et al., 2008; Myers et al., 2000; Perez et al., 2019; Tavşanoğlu & Pausas, 2018), and biased coverage of plant organs (toward leaf traits; (Cornwell et al., 2019; Kühn et al., 2021)) often hinder the use of less popular traits. This way, some of the valuable traits may be omitted due to their relatively low level of previous usage documented in the scientific literature, or limited availability. Therefore, it is not surprising that numerous studies simply include the sets of functional traits proposed in classic papers from the field (Díaz et al., 2016; Westoby, 1998; Wright et al., 2004), as this methodology already has been tried by numerous researchers. This often makes it a default approach (Coleman et al., 2020; Finegan et al., 2015; Hoffmann et al., 2005). However, every research question is different and covers at least a different group of species, and a different spatiotemporal scale. Taking into account the suggestions of de Bello et al. (2021), which concern careful, justified selection of traits for each study question, we may assume that for many research questions, those “default” trait sets are not the most well‐fitting ones to explain particular ecological mechanisms. At the same time, we recognize that the quantitative comparison of different trait sets can be problematic, especially for scientists who do not specialize in functional ecology and use the functional traits as a tool to solve varying research problems. Therefore, we decided to quantitatively compare the usefulness of different trait sets to present an example workflow that we hope could be useful to other researchers while exploring their datasets. We assumed that a good example dataset would be a data base representing a significant number of species with a high coverage of measurements of different traits. Then, we searched for a general research question, which would not determine the use of strictly limited sets of traits. Furthermore, when focusing on a more general research question, inclusion of traits representing diverse plant organs is particularly important. Here, we provide a quantitative comparison of the usefulness of different trait sets for predictions of species conservation status, as the fact of being threatened or not is determined by a complex set of factors that differs among species.

So far, there is a lack of quantitative comparisons of different trait sets. As mentioned above, numerous papers have presented the most optimal trait sets, widely used in ecological research (Díaz et al., 2016; Pierce et al., 2013; Westoby, 1998; Wright et al., 2004), but their results have never been compared. That is why we decided to study one of the crucial aspects of plant ecology in the context of changing climate, and to try to assess which traits will work best for predictions of which species could face the danger of being threatened. As a proxy of species threat, we used the conservation status of each species derived from the Red List and the Red Book of Ferns and Vascular Plants in Poland. Due to the low level of data coverage for herbaceous plants (Paź‐Dyderska et al., 2020), we decided to focus on woody species. Here, we provide a quantified comparison based on an extensive regional trait database, including all the woody species occurring in Poland (*N* = 387) together with 23 functional traits, both numeric and categoric, selected based on their availability in open databases. We evaluated the traits by preparing 13 different models based on previously conducted studies, and responding to a research question: How do models with different sets of functional traits predict the conservation status of different species? This was inspired by Miles (2020), who found that morphological traits are a highly explanative proxy to assess the risk of extinction for iguanian lizards, even in cases of data deficient or poorly known species. For plants, size and reproduction‐related traits also have high explanatory potential when predicting the probability of being threatened. For example, the recent study by Carmona et al. (2021) revealed that woody species (larger and with slowly adapting reproductive processes) show three times higher probability of being threatened when compared to herbaceous species (smaller and with more dynamic reproductive strategies). That is why in this approach we assumed that traits may explain the conservation status of the species.

In this study, we aimed to (1) determine the availability of functional traits representing different plant organs, (2) assess the trait sets with the highest explanatory potential, and (3) check whether model accuracy is enhanced by including a high number of traits. We hypothesized that (1) leaf traits are best represented both in terms of data availability and traits present in different models (Guerrero‐Ramírez et al., 2021; McCormack et al., 2017; Perez et al., 2019), (2) the trait sets with the highest explanatory potential will be those including traits evenly distributed across different plant organs (Kleyer & Minden, 2015), and (3) that including more traits would not lead to a better model performance (Lefcheck et al., 2015).

## 2 MATERIALS AND METHODS

### 2.1 Data collection

We prepared a list of 387 woody species occurring in Poland. To include the whole dendroflora, accounting for trees, shrubs, and subshrubs (i.e., species characterized by the lignification of only the lower parts of the shoots, species only weakly woody or species that are woody but survive only a few growing seasons), we compiled data from numerous sources (CABI, 2020; Kaźmierczakowa et al., 2014, 2016; Rutkowski, 2006; Wild et al., 2019; Zieliński, 1987, 2004). We included both native and non‐native species. Subsequently, we verified all the species names using the Global Biodiversity Information Facility (2020) and The Plant List (2020). After assessing the conservation status of the species studied using the Red List and the Red Book of Ferns and Vascular Plants in Poland (Kaźmierczakowa et al., 2014, 2016), we considered 36 species (i.e., 9.3% of all the species studied) to be threatened. Then, we compiled trait data (Tables 1 and 2) by joining TRY and BIEN data (Kattge et al., 2020; Maitner et al., 2018). We downloaded data for all traits that were available for our species. Then, we completed as much missing data as possible, searching for information in botanical handbooks, identification keys, and red lists of threatened species (CABI, 2020; Kaźmierczakowa et al., 2014, 2016; Rutkowski, 2006; Wild et al., 2019; Zieliński, 1987, 2004). These were mainly information on flowering onset, dispersal factors, leaf life span, nectar accessibility, and nectar amount. After completing the database, we decided to continue the analysis on the set of traits that were available for at least 25% of the species. As some parts of the trait values were still missing and we did not want to exclude less represented species, we performed data imputation based on correlations among traits and between traits and phylogeny (Pyšek et al., 2015), using an approach analogous to Dyderski and Jagodziński (2021). As previously stated, we only imputed data for the traits that had at least 25% completeness with measured values obtained from databases and other sources used (CABI, 2020; Kaźmierczakowa et al., 2014, 2016; Rutkowski, 2006; Wild et al., 2019; Zieliński, 1987, 2004). We imputed missing values using the random forest method from the missForest package (Stekhoven & Bühlmann, 2012) as recommended by Penone et al. (2014). We used the PVR package (Santos, 2018) to increase the predictive potential of imputation models using phylogenetic eigenvectors (Diniz‐Filho et al., 1998). According to the suggestion of Penone et al. (2014), we used the first 15 phylogenetic factors, which covered 63.9% of phylogenetic distance variation. The normalized root mean squared error of imputed traits was 0.4255. The phylogenetic tree was obtained from the megatree by Jin and Qian (2019) included in the V.phylo.maker package.

**TABLE 1:** Overview of the numerical traits. The values presented are from before the imputation of trait values.

Plant organ	Trait	Abbreviation	Completeness [%]	Unit	Mean	SD	Min	Max
Flower	Onset of flowering	OF	65.37	months	5.35	1.29	2.00	9.00
Stem	Height	H	65.89	m	7.25	8.06	0.04	36.39
Leaf	Leaf area	LA	46.51	cm^2^	27.14	38.82	0.04	289.22
Leaf	Leaf carbon	LC	26.36	g g^−1^	0.47	0.05	0.27	0.60
Leaf	Leaf carbon‐nitrogen ratio	CN	32.04	g g^−1^	22.19	11.02	8.41	77.42
Leaf	Leaf dry mass	LDM	46.77	g	0.23	0.48	0.00	3.65
Leaf	Leaf dry matter content	LDMC	42.64	g g^−1^	0.34	0.09	0.17	0.81
Leaf	Leaf nitrogen	LN	47.80	g g^−1^	0.0218	0.0076	0.0026	0.0533
Leaf	Leaf phosphorus	LP	39.53	g g^−1^	0.0018	0.0009	0.0002	0.0056
Seed	Seed dry mass	SM	64.34	g	0.39	1.66	0.01	14.56
Seed	Seed length	SL	27.65	cm	0.54	0.51	0.04	3.29
Leaf	Specific leaf area	SLA	57.88	cm^2^ g^−1^	168.54	77.58	22.19	507.91
Stem	Specific stem density	SSD	24.55	g cm^−3^	0.49	0.12	0.24	0.81

**TABLE 2:** Overview of the categorical traits.

Trait	Abbreviation	Completeness
Dispersal syndrome: animals	DS	99.48
Dispersal syndrome: autochory		99.48
Dispersal syndrome: human		99.48
Dispersal syndrome: water		99.48
Dispersal syndrome: wind		97.42
Leaf lifespan: less than 1 year	LLS	99.48
Leaf lifespan: 1 to 2 years		99.48
Leaf lifespan: more than 2 years		99.48
Nectar accessibility	NAC	99.48
Nectar amount	NAM	99.48

### 2.2 Data analysis

We performed all the analyses using the R software (R Core Team, 2020). We compared the performance of 13 models (Table 3) including different sets of functional traits according to the same methodological approach. We chose traits for the models named LHS, LES, Reich, Díaz, CSR, LeafStoich, and Morpho based on previous findings (Table 3). We developed models FlowerFruit, Seed, FlowFruitSeed, Leaf, and Stem by focusing on a given plant organ (or a certain function, in the case of the model focused on reproduction), to check their explanatory potential and to compare the predictive power of traits representing different organs (Kleyer & Minden, 2015). In the last model, we included all traits that we collected in this study. In terms of the selection of the models used in our study, we acknowledge that the best methodologically justified approach would have been to conduct a meta‐analysis of all functional trait‐related studies regarding species conservation and then select the most suitable trait sets for our study. However, due to the limited availability of trait measurements for the species we studied, we had to focus on the trait sets that used the available traits. The trait selection process was not conducted systematically, and the limited availability of traits forced us to choose the approach of including only a few examples of trait‐based models (Table 3).

**TABLE 3:** Overview of the models studied.

Approach	Model	Traits used	Rationale
Holistic concepts	LHS	SLA, height, seed mass	A classic leaf‐height‐seed (LHS) plant ecology strategy proposed by Westoby (1998)
	LES	Leaf nitrogen, SLA, leaf lifespan	Set of traits inspired by the concept of “Leaf Economics Spectrum” of Wright et al. (2004)
	Reich	Leaf nitrogen, leaf phosphorus, SLA	Set of traits inspired by the findings of Reich et al. (2010)
	Díaz	SLA, height, seed mass, leaf area, leaf nitrogen, specific stem density	The global plant trait spectrum proposed by Díaz et al. (2016), indicating major trade‐offs driving the growth and development of plants worldwide
	CSR	SLA, leaf area, leaf nitrogen, leaf carbon, LDMC, leaf dry mass	CSR classification (competitor/stress tolerator/ruderal) representing the fundamental trade‐offs between economics and size of plants, proposed by Pierce et al. (2013)
Traits selected for the biological function	LeafStoich	Leaf carbon, leaf carbon‐nitrogen ratio, leaf nitrogen, leaf phosphorus	Elemental composition traits. According to Kleyer and Minden (2015) relationships are stronger while within a given biological function.
	Morpho	Height, leaf area, LDMC, seed length, SLA, specific stem density, dispersal syndrome	Form traits. According to Kleyer and Minden (2015) relationships are stronger while within a given biological function
Traits selected for the organ represented	FlowerFruit	Onset of flowering, dispersal syndrome, nectar accessibility, nectar amount	Focus on the role of flower and fruit traits
	Seed	Seed mass, seed length, dispersal syndrome	Focus on the role of seed traits
	FlowFruitSeed	Onset of flowering, seed mass, seed length, dispersal syndrome, nectar accessibility, nectar amount	Focus on the role of the reproductive traits (flower, fruit, and seed traits)
	Leaf	Leaf area, leaf carbon, leaf carbon‐nitrogen ratio, leaf dry mass, LDMC, leaf nitrogen, leaf phosphorus, SLA, leaf lifespan	Focus on the role of leaf traits.
	Stem	Height, specific stem density	Focus on the role of stem traits
All the traits	All	Onset of flowering, height, leaf area, leaf carbon, leaf carbon‐nitrogen ratio, leaf dry mass, LDMC, leaf nitrogen, leaf phosphorus, seed mass, seed length, SLA, specific stem density, dispersal syndrome, leaf lifespan, nectar accessibility, nectar amount	All the traits from our trait data‐base

We decided to test the performance of each model by executing an exemplary research question: How do models with different sets of functional traits predict the conservation status of different species? To assess the predictive power of plant traits used in each model, we used a machine learning technique, as this approach enables signal extraction from datasets characterized by unbalanced classes (in our case threatened [1] vs. nonthreatened [0] species). Also, machine learning techniques allow for assessing the importance of each predictor, account for multiple interactions among variables, and are more robust to collinearity than other regression techniques (Breiman, 2001). We chose the random forest algorithm, which is based on multiple decision or classification trees, which leads to better stability and accuracy of the model obtained (Breiman, 2001). Random forest has been successfully applied for assessing the conservation status of habitats (Reynolds et al., 2016) or for predicting functional traits of trees (Dyderski & Jagodziński, 2019). We randomly selected 66.6% of observations used as a training set and the remaining 33.3% of observations as a test set, allowing for model performance validation. We used the caret::createDataPartition() function (Kuhn et al., 2020) to randomly split data into training and test datasets with equal proportions of positive and negative cases. We performed internal repeated cross‐validation (10 repeats, 10 times) to decrease model overfitting. To reduce the imbalance of classes (351 nonthreatened vs. 36 threatened species), we used the SMOTE (Synthetic Minority Over‐Sampling Technique) algorithm (Chawla et al., 2002) that simultaneously downscales the observations from the dominant class (nonthreatened species) and upscales the observations from the underrepresented class (threatened species). To increase the robustness of the models, we ran SMOTE with repeated cross‐validation subsampling.

### 2.3 Model evaluation

To assess accuracy of the models, we used area under the receiver‐operator curve (AUC), based on the confusion matrix of binary classification (ratio of true‐positive, false‐positive, true‐negative, and false‐negative observations). AUC ranges from 0 to 1 with the value 0.5 representing random selection (minimum model performance). AUC reflects the trade‐off between the sensitivity (true‐positive rate, i.e., ratio of correctly predicted positive records to all positive records) and specificity (true‐negative rate, i.e., ratio of correctly predicted negative records to all negative records) of a model. Accuracy is true‐positive and true‐negative records divided by the number of all records, which is the proportion of correct predictions. Balanced accuracy takes uneven coverage of classes into consideration, as it artificially compensates the disproportion between the two groups of the species studied (threatened/unthreatened). Kappa represents the performance of the classifiers used by providing information about the agreement of the model prediction with the measured data. We decided to choose the best models according to their AUC values, as AUC is a parameter that evaluates the model output quantitatively and holistically.

## 3 RESULTS

### 3.1 Are the traits of different plant organs similarly available?

Categorical traits had the highest completeness (Table 2): dispersal syndrome, leaf life span, nectar accessibility, and nectar amount (99.5%, with the exception of dispersal syndrome: wind, with 97.4% coverage). Numerical traits of the highest completeness (Table 1) were as follows: height (65.9%), onset of flowering (65.4%), and seed mass (64.3%). The mean completeness of leaf‐related, reproduction‐related, and stem‐related numerical traits was 42%, 52%, and 45%, respectively. As the completeness of the categorical traits was almost the same for all the traits and amounted to over 97%, we did not include them in this comparison of completeness differences among various organs. Leaf‐related traits were the most numerous (11 of 23 traits), and also were most often used in the models, as they occurred in nine of 13 models (Table 3). There were eight reproduction‐related traits (joined traits of flowers, fruits, and seeds), and two stem‐related traits, which occurred in seven and four out of 13 models, respectively.

### 3.2 Which trait sets have the highest explanatory potential?

**FIGURE 1:**
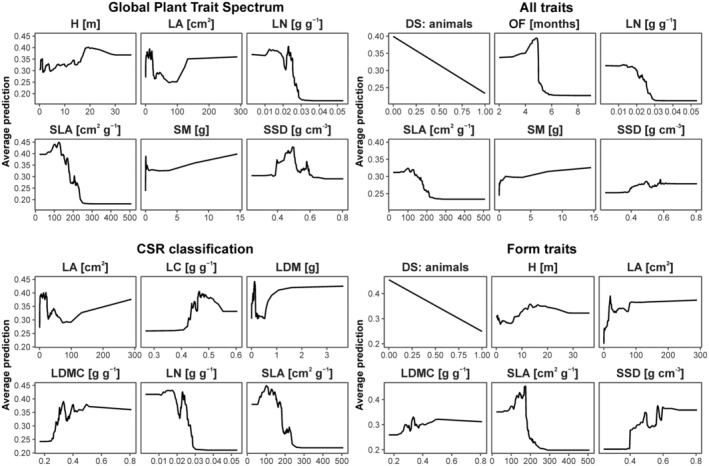
Partial dependence plots for the six most important traits of the four best models (from the top left corner: model Díaz, All, CSR, and Morpho. A higher average prediction indicates a higher probability that a given species is threatened, according to a given model. We reduced the number of traits presented to only the six most important ones to maintain clarity.

The models with the highest AUC (Table 4) were Díaz (0.996), All (0.995), CSR (0.993), and Morpho (0.991). Their accuracy amounted to 0.881, 0.894, 0.822, and 0.894, and their balanced accuracy to 0.935, 0.942, 0.902, and 0.942, respectively. In model Díaz, the most important traits were seed mass, specific leaf area (SLA, leaf area to dry mass ratio), and specific stem density; in model All—dispersal syndrome: animals, onset of flowering, and seed mass, in model CSR—leaf nitrogen, SLA, and leaf carbon, and in model Morpho—SLA, leaf area, and dispersal syndrome: animals. Partial dependence plots for the six most important traits of the four best models (Figure 1) indicated that the performance of the same traits across different models was similar (e.g., SLA, leaf area, and leaf nitrogen).

**TABLE 4:** Overview of the model outputs.

Model	LHS	LES	Reich	Díaz	CSR	LeafStoich	Morpho	FlowerFruit	Seed	FlowFruitSeed	Leaf	Stem	All
Confusion matrix													
True positive	36	22	36	36	36	36	36	18	36	36	36	36	36
False positive	59	87	71	46	69	50	41	62	39	47	57	71	41
True negative	292	264	280	305	282	301	310	289	312	304	294	280	310
False negative	0	14	0	0	0	0	0	18	0	0	0	0	0
Accuracy	0.848	0.739	0.817	0.881	0.822	0.871	0.894	0.793	0.899	0.879	0.853	0.817	0.894
Kappa	0.479	0.190	0.423	0.552	0.432	0.528	0.585	0.209	0.598	0.546	0.490	0.423	0.585
Sensitivity	0.832	0.752	0.798	0.869	0.803	0.858	0.883	0.823	0.889	0.866	0.838	0.798	0.883
Specificity	1.000	0.611	1.000	1.000	1.000	1.000	1.000	0.500	1.000	1.000	1.000	1.000	1.000
Balanced accuracy	0.916	0.682	0.899	0.935	0.902	0.929	0.942	0.662	0.944	0.933	0.919	0.899	0.942
AUC	0.967	0.766	0.967	0.996	0.993	0.975	0.991	0.798	0.982	0.984	0.986	0.949	0.995
Variable importance													
CN						0.120					0.050		0.012
DS: animals							0.023	−0.022	0.036	0.026			0.029
DS: autochory							0.001	0.000		0.001			0.001
DS: human							0.003	0.002		0.004			0.000
DS: water							0.001	0.002		0.000			0.003
DS: wind							0.005	−0.022		0.014			0.014
OF								0.000		0.080			0.020
H	0.065			0.030			0.019					0.109	0.007
LA				0.019	0.012		0.030				0.005		0.008
LC					0.017	0.082					0.015		0.009
LDM					0.012						0.008		0.006
LDMC					0.008		0.020				0.007		0.006
LLS: <1 year		0.009									0.003		0.001
LLS: >2 years		0.000									0.000		0.000
LLS: 1–2 years		0.007									0.000		0.000
LN		0.012	0.076	0.031	0.039	0.099			0.035		0.018		0.016
LP			0.059			0.073			0.023		0.023		0.007
NAC													0.001
NAM													0.002
SL							0.018		0.027	0.046			0.005
SLA	0.092	0.021	0.075	0.029	0.035		0.046		0.026		0.024		0.018
SM	0.049			0.039						0.081			0.018
SSD				0.031			0.022		0.032			0.130	0.016

In model All, where the comparison of variable importance was possible (as it includes all the traits studied), the trait of the highest importance was dispersal syndrome: animals (0.029), followed by onset of flowering (0.020), seed mass, and SLA (0.018). Traits of the lowest importance were leaf life span: more than 2 years, leaf life span: 1 to 2 years, and dispersal syndrome: human (≥0.001).

### 3.3 Does model accuracy increase with the number of traits used?

The highest AUC was in model Díaz that included only six traits (Table 4). However, model All, which included all 23 traits, provided the second highest AUC value. The next models with the highest AUC scores were CSR and Morpho, which included six and 11 traits, respectively. Model CSR, however, had lower values for the remaining parameters than the other three best models.

On the contrary, the rest of the models that included more than the median number of all traits (i.e., with more than six traits used) did not have equally satisfactory performance. Although the model with the worst general performance was LES, which included five traits and had the lowest values for four of six parameters analyzed, model FlowerFruit (including eight traits) had the lowest values of balanced accuracy and specificity and second lowest AUC value.

Three of the models studied included traits representing all the aboveground organs of a plant: models Díaz, Morpho, and All, all of which were among the four best models. Model Díaz and second model All had the highest AUC values (0.996 and 0.995, respectively). The AUC of model Morpho with the value of 0.991 was fourth among all of the models studied. However, in the cases of these three models, values of the rest of the parameters studied were high, which, together with very high AUC values, gave them the highest rankings.

## 4 DISCUSSION

Our findings suggest that what matters most for a trait set of high explanatory power is not the number of traits included, but rather a precise selection of those traits, which is in line with the findings of Lefcheck et al. (2015), who similarly stated that more is not always better. We found no evidence that including more traits in the model will lead to higher model accuracy. Also, the models that include the traits representing all plant organs do not necessarily outrank models that include, for example, only leaf‐related traits, in terms of their AUC score. Differences among models can result from the correlated structure of functional traits (Díaz et al., 2016; Kleyer et al., 2019; Wright et al., 2004). That way, including one or more aspects (traits) of a particular life strategy in the model can split the variability into more specific responses, or account for general trade‐offs of resource utilization strategies. For example, leaf nitrogen and SLA are strongly correlated, as they reflect higher investment into leaf acquisition than leaf persistence (Díaz et al., 2016; Wright et al., 2004). Therefore, including both of them can provide deeper insight into both mechanisms (structural defense connected with leaf mechanical structure versus nitrogen utilization for photosynthesis efficiency), as in model Díaz. However, interactions between them can affect response curves. In contrast, accounting for one of these traits can provide a clearer response curve, but not accounting for such complexity (Figure 1. “Form traits”). Therefore, the selection of traits requires a clear conceptualization of the study aim and desired level of complexity at the trait selection stage.

According to previous studies from the field, our results show that leaf traits can provide high accuracy model outputs (Hoffmann et al., 2005; Martínez‐Garza et al., 2005; Moravcová et al., 2015), and can even outperform models including traits of a larger number of plant organs (in our case, model CSR had higher AUC than model Morpho). In the case of our findings, two of the three best models included traits representing all aboveground plant organs, and the third one only included leaves. This might be an outcome of overrepresentation of studies focusing on leaf traits (Kühn et al., 2021), which can result in a better coverage of leaf traits with measurements and their higher explanatory potential. However, model CSR provided lower values of the remaining parameters (besides AUC), which leads to the conclusion that models including traits representing a higher number of plant organs provide better results. Thus, based on the output of those three models (i.e., Díaz, Morpho, and All), we can suggest that using carefully selected traits covering more plant organs is highly beneficial for model performance (Kleyer & Minden, 2015; Lefcheck et al., 2015). In the case of our study, Model Díaz, which reached the highest AUC, included traits with the mean completeness of 51%, whereas the mean completeness of all numeric traits studied was 45%. This 6% higher completeness can be related to the proven explanatory potential of the traits included in model Díaz et al. (2016). Simultaneously, this may be because the “popular” traits are measured more often and subsequently are better covered in databases (Rosado et al., 2013). Thus, some traits of high explanatory potential may be overlooked. This could be suggested by the high values of parameters in model All, which uses both widely used (e.g., height, leaf nitrogen, seed mass, and SLA) and less known (or less available) traits (such as dispersal syndrome, nectar accessibility, nectar amount). Trait data availability is also related to the quality of research that ecologists focusing on functioning of plants and their responses to climate change can conduct. For example, based on a systematic global review, Kühn et al. (2021) revealed a consistency among traits that best identified positive responses in plants to climate and environmental changes, including lower or higher SLA, lower or higher plant height, greater water‐use efficiency, greater resprouting ability, lower relative growth rate, greater clonality/bud banks/belowground storage, higher wood density, and greater rooting depth. Taking into consideration that those traits have been carefully selected based on 148 studies from all over the world, their potential in research on coping mechanisms of plants is highly significant. However, the ongoing, problematic issue of insufficient coverage of species with trait measurements hinders their immediate use in ecological studies. We come to a point where the traits of highest potential have been identified, but due to their low availability, we probably have to follow two paths simultaneously: work harder toward filling as many gaps in the trait databases as possible; and use trait data that are currently available in the best possible way.

Although our study provides a valuable quantification of frequently used model outputs, it still has some considerable drawbacks. First, we tested data collected only for one country and only for woody plants. Omitting the herbaceous species could impact the results (Gilliam & Roberts, 2003). However, as we wanted to compare models using different traits for the same set of species, we do not consider this a significant shortcoming. Second, we compiled a dataset of only 23 traits, while there are hundreds of usable traits in databases (Enquist et al., 2016; Guerrero‐Ramírez et al., 2021; Kattge et al., 2020). Yet, as we mentioned in the Methods section, in the final version of our database, we included only those traits for which completeness was at least 25%. As we had to exclude numerous traits from our database, a side effect of our work was once again underlining the need for thorough work on further supplementing databases with data (Cornwell et al., 2019). Third, of the 23 traits used in our study, none represented the underground plant organs. As belowground plant traits are crucial for a fuller understanding of plant strategies (Bardgett et al., 2014; Iversen et al., 2017), we admit that not including root traits in our database is a significant problem (Kleyer & Minden, 2015). However, this drawback derives strictly from the poor root data coverage of the species set studied. This field of functional ecology is developing dynamically (Guerrero‐Ramírez et al., 2021), yet the coverage of species with measurements requires time. Moreover, the classic trait sets were often developed and published some years ago (Díaz et al., 2016; Pierce et al., 2013; Westoby, 1998); thus, they often do not include root traits, as data coverage at the time of publication was even more scarce than it is today. Fourth, we tested only one exemplary research question, and possibly modeling based on a different research question could lead to different results, as the explanatory potential of different traits may vary for different hypotheses. Modeling the conservation status of a species was, however, a convenient variable used in our study, as it was easily verifiable for all of the species. Lastly, we compared 13 trait sets, although we know that there are numerous propositions of trait sets (Czortek et al., 2018; Jagodziński et al., 2016). Study designs representing differentiated study aims often combine traits into sets that, according to expert knowledge or previous research, are the most appropriate to answer a given research question (Aguirre‐Gutiérrez et al., 2016; de Bello et al., 2021; Petchey & Gaston, 2006). Therefore, due to their specificity, they often are not universal. We, on the contrary, aimed to focus on sets of functional traits that included less specific traits with significant explanatory potential for a wide range of research questions.

## 5 CONCLUSIONS

Here, we contribute to the development of guidelines on completing the optimal trait set for a given study. Primarily, as models based on trait sets proposed in previous studies had relatively better output in our study (Díaz et al., 2016; Pierce et al., 2013; Westoby, 1998), we recommend selecting potentially beneficial traits based on previous findings. Traits representing different plant organs should be included in the models whenever possible (Kleyer & Minden, 2015; Lefcheck et al., 2015), as three of the four best models from our comparison included traits of all aboveground organs. Using, for example, only leaf traits can also provide relatively good results, as the third best model only used leaf traits (Pierce et al., 2013). However, the high explanatory power of leaf traits can derive from high completeness of those traits: We should focus on improving the coverage of trait databases with new data, because this effort may enable the use of further, possibly powerful traits (Cornwell et al., 2019). Including numerous traits can also lead to improvement of model output, but the completeness of the traits should be as high as possible to capture their interspecific variability (Paź‐Dyderska et al., 2020). For studies where the research questions are not highly specific, we recommend using the trait set from model Díaz (i.e., SLA, height, seed mass, leaf area, leaf nitrogen, and specific stem density), as it showed the best results using only six traits (Díaz et al., 2016), which we believe is a favorable trade‐off for use in further ecological modeling research.

## AUTHOR CONTRIBUTIONS

**Sonia Paź‐Dyderska:** Conceptualization (equal); data curation (lead); formal analysis (lead); investigation (lead); methodology (equal); software (lead); validation (equal); visualization (lead); writing – original draft (lead). **Andrzej M. Jagodziński:** Conceptualization (equal); methodology (equal); supervision (lead); validation (equal); writing – review and editing (lead).

## FUNDING INFORMATION

The study was supported by the Institute of Dendrology, Polish Academy of Sciences, Kórnik, Poland.

## CONFLICT OF INTEREST STATEMENT

The authors declare no conflict of interest. The funders had no role in the design of the study; in the collection, analyses, or interpretation of data; in the writing of the manuscript, or in the decision to publish the results.

## Data Availability

Datasets will be publicly available when the article is published. For now, data are available in the FigShare repository using this private link (for review only): https://doi.org/10.6084/m9.figshare.12961847
